# Acceptability of Newborn Screening for Sickle Cell Disease Among Pregnant Women in Bukavu, Democratic Republic of the Congo: Factors Associated With Uptake and Implications for Public Health

**DOI:** 10.1155/anem/3189576

**Published:** 2025-05-06

**Authors:** Nash Mwanza Nangunia, Olivier Mukuku, Viviane Bianga Feza, Yves Mulindilwa Kyembwa, Théophile Barhwamire Kabesha, André Kabamba Mutombo, Stanislas Okitotsho Wembonyama

**Affiliations:** ^1^Faculty of Medicine, Official University of Bukavu, Bukavu, Democratic Republic of the Congo; ^2^Institut Supérieur des Techniques Médicales de Lubumbashi, Lubumbashi, Democratic Republic of the Congo; ^3^Health Economics and HIV and AIDS Research Division, University of KwaZulu-Natal, Durban, KwaZulu-Natal, South Africa; ^4^Faculty of Medicine, Official University of Mbuji-Mayi, Mbuji-Mayi, Democratic Republic of the Congo; ^5^Faculty of Medicine, University of Lubumbashi, Lubumbashi, Democratic Republic of the Congo

**Keywords:** acceptability, Bukavu, newborn screening, sickle cell disease

## Abstract

**Introduction:** Sickle cell disease (SCD) is a serious genetic disorder, often diagnosed early, which can lead to significant complications. Although newborn screening (NBS) for SCD is an effective intervention for reducing the impact of SCD in developed countries, it remains poorly accessible in sub-Saharan Africa, where the disease is particularly prevalent. This study assessed the acceptability of NBS and the factors influencing it among pregnant women in Bukavu, in the Democratic Republic of the Congo.

**Methods:** A survey of pregnant women in Bukavu was conducted between December 1, 2023, and January 31, 2024. Data were collected using a semi-structured questionnaire covering sociodemographic characteristics, knowledge, and attitudes toward NBS. Multiple logistic regression was used to identify factors associated with NBS acceptability.

**Results:** Out of a total of 350 respondents approached, 300 voluntarily agreed to answer our questionnaire, resulting in a response rate of 85.7%. Among them, the acceptability rate of NBS was 80.0%. Logistic regression analysis indicated that recognizing SCD as a blood disorder was strongly linked to the acceptability of NBS (adjusted OR = 2.6; 95% CI [1.4–4.9], *p*=0.002). In addition, individuals who were aware that SCD could be diagnosed at any point in life were more inclined to accept NBS (adjusted OR = 2.0; 95% CI [1.1–3.8], *p*=0.024). There were no significant associations observed with age, marital status, educational level, professional occupation, religion, knowledge of electrophoretic status, and awareness that SCD can be diagnosed in the neonatal period, or awareness that SCD can be diagnosed at any other time in life.

**Conclusion:** This study demonstrates a significant level of acceptability of NBS among pregnant women in Bukavu, which is influenced by their understanding of SCD and knowledge about diagnostic possibilities. Implementing awareness-raising initiatives focused on key topics, such as the benefits of NBS, the implications of early diagnosis, the availability of follow-up care, increasing knowledge about SCD as a blood disorder, and its potential for diagnosis at any stage of life, could further enhance acceptability.

## 1. Introduction

Sickle cell disease (SCD), or sickle cell anemia, is a genetic disorder caused by an abnormality in hemoglobin (Hb), transmitted in an autosomal recessive manner. The disease results in the production of Hb S (HbS), which deforms red blood cells, causing vascular obstructions and a range of health problems [1]. The lack of reliable data on SCD in many countries makes it difficult to estimate its prevalence. Estimates show significant variations in the distribution of the S allele, particularly in sub-Saharan Africa (SSA) (2%–38%), India (17%–30%), and the eastern Mediterranean (Saudi Arabia [1% to 29%] and Iraq [0% to 22%]) [2–4]. In the Democratic Republic of the Congo (DRC), recent epidemiological data indicate that 2% of newborns are homozygous for HbS, with an estimated 40,000 sickle cell births each year [5, 6]. These children face high mortality, with 50%–90% dying before the age of 5 because of delayed diagnosis and limited resources [7, 8].

SCD is a significant contributor to infant mortality in developing nations, where newborn screening (NBS) is frequently lacking. The efficacy of NBS in reducing this mortality hinges on its acceptability, which is impacted by various sociocultural factors [9–11]. In SSA, the condition is occasionally viewed as a curse or attributed to witchcraft, affecting perceptions toward treatment [9, 12, 13]. In developing countries, NBS is frequently postponed and only conducted after an initial crisis, leading to delayed diagnosis [14].

Although NBS pilot projects have been initiated in SSA, a national program has not been established, despite external funding [7, 15–18]. These efforts have faced challenges, particularly regarding the acceptability of NBS by families, healthcare professionals, and authorities. In light of this, some researchers advocate for a bottom-up approach to foster community backing prior to the rollout of public health initiatives [19].

With this in mind, the aim of this study is to assess the acceptability of NBS among pregnant women in Bukavu, in the DRC, and to identify the factors associated with their uptake of this intervention. The study focuses on the impact of knowledge about SCD and the diagnostic options available, in order to gain a better understanding of the determinants of acceptability of NBS. The results will provide essential elements for the implementation of targeted awareness-raising strategies, which could specifically be tailored to the cultural and socioeconomic context of the Bukavu region. These findings will also help guide the development of local public health policies in Bukavu, particularly in integrating NBS into regional healthcare services, and will support the broader integration of NBS into nationwide public health programs in the DRC.

## 2. Materials and Methods

### 2.1. Study Framework

This study was conducted in Bukavu, a city in the east of the DRC that serves as the capital of the South Kivu province. Formerly known as Costermansville, Bukavu is situated on the south-western shore of Lake Kivu. As of 2024, the population of Bukavu is estimated to be 1,308,470, a substantial increase from the 1950 population of 16,296. The city experiences an annual growth rate of 4.78%, resulting in an approximate addition of 59,690 residents in the past year [20]. Bukavu spans an area of 6000 ha, equivalent to 60 km^2^, with a population density of 21,808 inhabitants per square kilometer.

Bukavu is a cosmopolitan urban area that unites various ethnic groups, with the majority being of Bantu origin. Because of its strategic location and cultural diversity, the city serves as a hub for the economic, social, and administrative activities of the province.

### 2.2. Description of the Study

A cross-sectional survey study was conducted among pregnant women, irrespective of their gestational age, sequentially selected during their antenatal visits in different referral health centers across the three communes of Bukavu city. The survey was conducted between December 1, 2023, and January 31, 2024.

Cochran's formula for calculating sample size in descriptive studies (*n* = z^2^pq/d^2^) was utilized [21], with *z* representing the normal standard deviation (SD) for a 95% confidence interval (1.96); *p* denoting the estimated prevalence of SCD (5.01%) [22]; *q* as 1—*p* (0.9499); and *d* indicating the margin of error (0.025). The minimum sample size required was therefore 293 participants, which was inflated to 345 to account for a nonresponse rate of 15%.

### 2.3. Data Collection and Tools Used

A semi-structured questionnaire, previously utilized in similar studies [23, 24], was developed to gather data on participants' sociodemographic characteristics, knowledge of NBS, attitudes toward NBS, and perceptions of NBS and diagnostic options. The questionnaire comprised closed questions with response choices of “Yes,” “No,” or “Don't know,” with no elaboration encouraged for the closed-ended questions. The study variables were categorized into different groups. Sociodemographic factors included age, marital status, education, occupation, and religion. Age groups were defined as under 25, 25–29, 30–34, and 35 years or older. Education levels were classified as primary, secondary, and higher/university. Regarding occupation, participants were grouped as business, employed, or unemployed. Lastly, religions were divided into Protestants/Pentecostals, Catholics, and Muslims. Participants' knowledge of SCD was assessed through closed questions regarding the nature of the disease, transmission, and diagnostic methods. Questions included statements like “Sickle cell disease is a blood disorder” and “Sickle cell disease is contagious,” with response options of “true” or “false.”. Other inquiries delved into understanding the modes of transmission, such as heredity (from single mother, single father, or both parents), and the feasibility of prenatal, neonatal, or later-life diagnosis. Attitudes toward NBS were evaluated based on acceptance of screening before marriage, during pregnancy, before childbirth, and after having children. Participants were also questioned about their willingness to screen their spouse and their reasons for or against screening. Response choices for these queries were “yes,” “no,” or “I don't know.” Reasons for acceptance or refusal were gathered from open-ended responses and categorized as either “for” or “against” screening.

Three teams of six interviewers and a doctor were formed to administer the questionnaires in French and Swahili (the local language). If a participant could not read or write, the interviewer completed the questionnaire for her after receiving her answers. Before data collection, a pilot study was conducted with 10 participants to assess their comprehension of the questions and make any necessary adjustments to the questionnaire. The pilot interviews aimed to ensure that no questions were ambiguous and determine the approximate length of the interviews, which averaged 25 min. Data from the pilot interviews were not included in the final results.

The interviewers underwent 3 days of training in interview techniques, the study's objectives, and ethical considerations. The principal investigator and supervisors closely monitored data collection daily and reviewed questionnaires for accuracy and coherence.

The survey was conducted between December 1, 2023, and January 31, 2024.

This study was carried out in primary healthcare facilities located in the Ibanda municipality, including Panzi General Referral Hospital, CHAI Hospital Center, Ruhigita Clinic, and CELPA Hospital Center. All interviews were conducted face-to-face with pregnant women attending antenatal care clinics within these facilities. The participants were approached and interviewed on-site after obtaining their informed consent. No home visits were conducted for the purpose of this study.

### 2.4. Data Analysis

Data were entered and checked in Excel 2019, then analyzed using STATA 16 software. Sociodemographic characteristics were analyzed using descriptive statistics. Chi-square tests were used to examine the association between sociodemographic variables and survey items regarding knowledge of NBS with acceptability of NBS. Variables with a *p* value of less than 0.2 in the chi square analysis were included in multiple logistic regression models to identify factors independently associated with acceptability of NBS. Overall statistical significance was defined as *p* < 0.05.

### 2.5. Ethical Considerations

The study was approved by the Medical Ethics Committee of the University of Bukavu (Approval No.: UOB/CEM/010/2023). Authorization to conduct the study was also obtained from the Provincial Health Division of South Kivu Province (N°061/CD/DPSSK/2024). Community consent was obtained from the municipal authorities of the three communes. All participants signed an informed consent form before participating in the study. Confidentiality of the collected information was ensured, and participation was entirely voluntary. Participants had the right to withdraw from the study at any time without facing any consequences.

This study was reported in accordance with the Strengthening the Reporting of Observational Studies in Epidemiology (STROBE) guidelines (see Supporting Information) (available here).

## 3. Results

Out of a total of 350 respondents approached, 300 voluntarily agreed to answer our questionnaire, resulting in a response rate of 85.7%. The data in Table 1 show that the mean (SD) age of respondents was 28.7 ± 5.7 years, with 60.3% under 30 years of age. In relation to marital status, 83.3% were married, and 16.7% were single. Nearly half of the respondents had completed secondary education (45.0%) or higher/university education (44.7%), while 10.3% had primary education. Regarding professional occupation, 51.3% of respondents were engaged in commerce, 19.7% were employees, and 29.0% did not have a professional occupation. The distribution of religious affiliation was almost equal between Protestants/Pentecostals (41.7%) and Catholics (41.0%), with 17.3% identifying as Muslims (Table 1).

Overall, among the 300 pregnant women surveyed, there was a relatively good level of knowledge about SCD. Approximately, 208 respondents (69.3%) correctly identified SCD as a blood disorder, and 213 respondents (71.0%) understood that it is inherited from both parents. However, some confusion was noted, with 58 (19.3%) and 57 (19.0%) respondents incorrectly believing it is inherited solely from the mother or father, respectively. The majority of respondents (255, 85.0%) were aware that SCD is not contagious.

**Figure 1:**
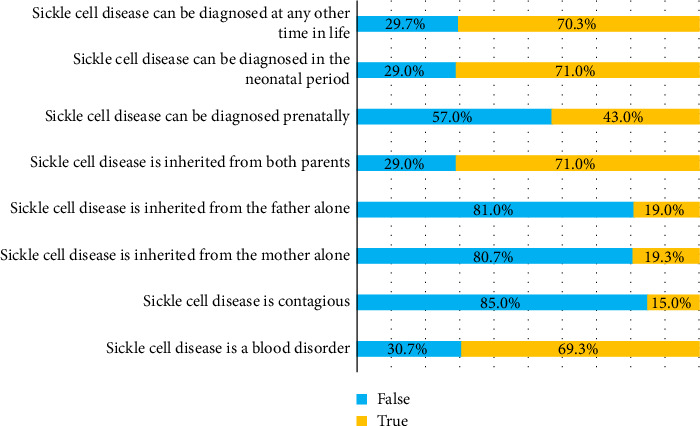
Respondents' (*N*=300) general knowledge of sickle cell disease.

In terms of diagnosis, 213 respondents (71.0%) were aware that SCD can be detected at birth (neonatal diagnosis), and 211 respondents (70.3%) were aware that it can be diagnosed at any stage of life. However, only 129 respondents (43.0%) were aware that prenatal diagnosis is possible, indicating a need for greater awareness of this issue (Figure 1).

Twenty-eight respondents (9.3%) knew their status in relation to SCD. Of these, one indicated that she had SCD (Hb SS), and two were carriers of the sickle cell trait (Hb AS).

**Figure 2:**
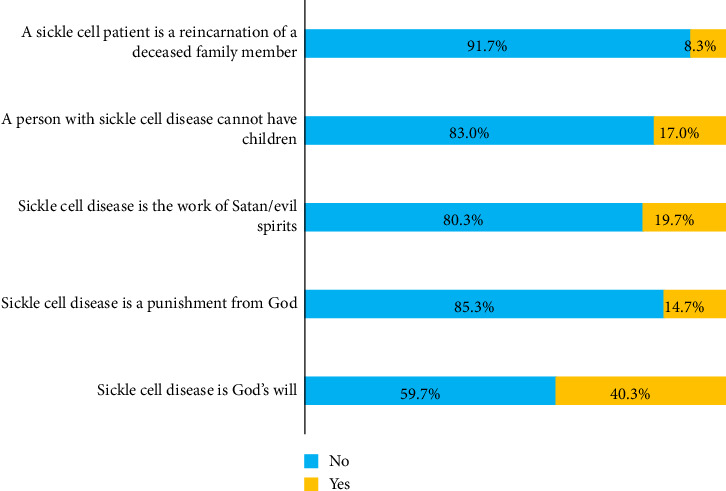
Beliefs of respondents (*N*=300) about sickle cell disease.

Analysis of the cultural perceptions and beliefs relating to SCD found that 40.3% of the participants (121 out of 300) considered that SCD was God's will. A smaller proportion (14.7%, or 44 participants) perceived SCD as a divine punishment, and 19.7% (59 participants) associated the disease with the work of Satan or evil spirits. Regarding reproductive capacity, 17.0% (51 participants) thought that a sickle cell patient could not have children. Finally, 8.3% (25 participants) believe that SCD is a reincarnation of a deceased family member (Figure 2).

**Figure 3:**
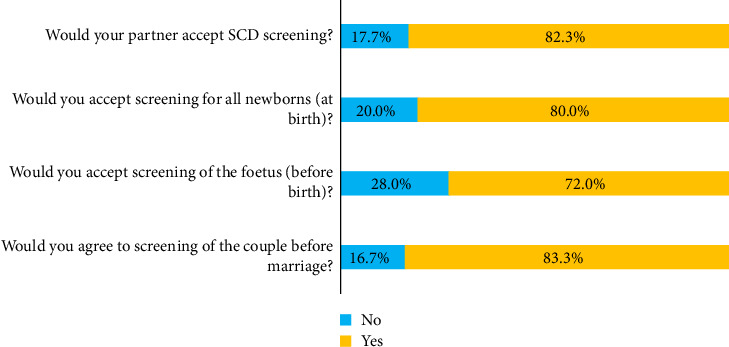
Attitudes of respondents (*n*=300) toward sickle cell screening.

Figure 3 indicates that the 300 pregnant women surveyed were predominantly supportive of screening for SCD. A majority (83.3%, or 250 participants) expressed willingness to undergo screening as a couple before marriage. In terms of prenatal fetal screening, 72.0% (216 participants) were in favor. When considering their partner's perspective, 82.3% (247 participants) believed that their partner would be supportive of NBS. Finally, NBS, i.e., screening all newborns at birth, was also well received, with 80.0% (240 participants) accepting it. Among those in favor of NBS (*n* = 240), a significant majority (202 women, 84.2%) justified their choice by the simple desire to know the state of their baby's health. A few participants were motivated by already having a child with SCD (17 women, 5.7%) or by the fact that NBS was free (14 women, 4.7%). Some emphasized the importance of NBS due to their carrier status and/or that of their spouse (seven women, 2.3%). The most common reasons cited for not undergoing NBS included the perceived need for spousal authorization (26 women, 8.7%) and the perceived lack of effective treatment for the disease (15 women, 5.0%). Some expressed fear of knowing their baby's sickle cell status (9 women, 3.0%) or concerns about potential harm from the injection (5 women, 1.7%). In addition, a few considered NBS unnecessary because of their Hb AA genotype and that of their partner (5 women, 1.7%).

Table 2 shows the association between acceptability of NBS and sociodemographic characteristics and knowledge of the respondents. Age, marital status, level of education, occupation, and religion showed no significant associations with acceptability of NBS.

NBS acceptability rates were consistent across age groups, with approximately 79% of the respondents in favor of NBS, and no statistically significant difference. Single women (88.0%) exhibited a higher acceptance rate compared to married women (78.4%), although this was not significant. In terms of education, respondents with a primary school level of education (67.7%) showed a slightly lower acceptance rate than those with secondary (81.5%) or higher/university education (81.3%), but this difference was not statistically significant.

Women who were unemployed or employed (85.1% and 84.8%, respectively) showed higher acceptability rates for NBS compared to those in commerce (75.3%), but this distinction was not statistically significant. Regarding religion, Catholics (81.3%) and Protestants (82.4%) exhibited greater acceptability rates than Muslims (71.2%), although this was not statistically significant.

Knowledge of electrophoretic status showed a tendency toward higher acceptability of NBS among respondents who knew their status (85.7% versus 79.4%), but this difference was not significant. On the other hand, those who were aware of the blood nature of SCD, its neonatal diagnosis, or that it could be diagnosed at any other time in life had significantly higher acceptability rates (85.6%, *p*=0.0005; 83.1%, *p*=0.036; and 83.9%, *p*=0.009, respectively).

The results of the multivariable logistic regression model show that two explanatory variables are significantly associated with the acceptability of NBS (Table 2). Firstly, respondents who recognized SCD as a blood disorder were 2.6 times more likely to accept screening, compared with those who did not (adjusted OR = 2.6; 95% CI [1.4–4.9]; *p*=0.002). Secondly, respondents who were aware that SCD could be diagnosed at any other time in life were 2.2 times more likely to accept NBS (adjusted OR = 2.0; 95% CI [1.1–3.8]; *p*=0.024).

## 4. Discussion

The acceptability of NBS is a key factor in the implementation of effective public health strategies. In our study conducted in Bukavu, the acceptability rate of NBS was found to be 80%, indicating an overall interest in this initiative among the target population. This rate is slightly lower than that reported by Katamea et al. [10] in Lubumbashi (DRC), where acceptability rate was 84.5%. However, both studies show a generally favorable trend toward the introduction of NBS in the country. It should be noted that the rates observed are comparable to those of studies conducted in other African countries such as Liberia and Nigeria, where NBS acceptability reached high values of 99% and 99.7%, respectively [25, 26].

The results of our study reveal that 69.3% of respondents identify SCD as a blood disorder, an essential factor for a better understanding of NBS and for encouraging participation in NBS. Although this figure is relatively high, it is still insufficient, and more education about the disease is needed. On the other hand, in the study by Katamea et al. [10] in Lubumbashi, 77.7% of participants had a good knowledge of SCD as an inherited blood disorder, which also reflects an encouraging level of awareness. This perception is crucial in encouraging acceptance of screening. However, only 18.55% of participants knew their own sickle cell status. Although this figure is higher than that observed in Nigeria (3.5%) [24], it highlights the underutilization of NBS in the Congolese population. These results highlight the need for greater awareness and better education on the importance of NBS, especially in high-prevalence areas such as the DRC [27].

The results of our study align with those of Babalola et al. [28] in Nigeria, who also found that knowledge and perceptions of SCD are significantly associated with the acceptability of NBS. Similar to our findings, mothers with a better understanding of the disease were more inclined to accept NBS. In our analysis, respondents who identified SCD as a blood disorder were 2.6 times more likely to accept NBS (adjusted OR = 2.6; 95% CI [1.4–4.9]; *p*=0.002), underscoring the importance of health education in promoting SCD.

The results of Babalola et al. [28] also suggest that mothers who believe that SCD can be diagnosed from the neonatal period are more likely to accept NBS, a finding that we also found. In our study, those who were aware that SCD could be diagnosed at any other time in life were 2.0 times more likely to accept NBS (adjusted OR = 2.0; 95% CI [1.1–3.8]; *p*=0.024). This finding highlights the importance of raising awareness of the benefits of NBS, including the possibility of detecting the disease early in life. These results underline that education and awareness of SCD, as well as the dissemination of information about NBS, are crucial levers for improving the acceptability of NCD, as studies both in Nigeria [28] and in our own context have shown.

The results of our study showed that erroneous beliefs about SCD, particularly its supernatural origin, remain an obstacle to the implementation of NBS programs. Supernatural explanations for the cause of SCD are common in many African contexts [24, 29]. Information campaigns aimed at demystifying these beliefs, using the media and community workers, would be beneficial. In addition, the involvement of religious leaders in raising awareness could play an important role, as suggested by the study by Katamea et al. [10], where religion was identified as a significant factor affecting the acceptability of NBS (*p* < 0.001).

With regard to the reasons for or against NBS, our study revealed several factors associated with the acceptability of NBS. Among participants in favor of NBS, most (67.33%) justified their choice by the simple desire to know the state of health of their baby. This response shows that early information about the health of the newborn is a determining factor in the acceptability of NBS, which is in line with the results of other studies that emphasize the importance of NBS for early management [24, 25]. In addition, 5.67% of women were motivated by the experience of already having an SCD child, illustrating increased awareness of the risk of the disease and the importance of early detection of new cases. Free NBS was also a motivating factor for 4.67% of participants, highlighting the impact of free NBS on the uptake of public health programs, particularly in low-resource settings. Finally, 2.33% of women stressed the importance of NBS because of their status as carriers of SCD, as well as that of their spouse, emphasizing the need to make couples more aware of their genetic status to avoid the transmission of the disease.

On the other hand, among the reasons given for not undergoing NBS, the most frequently cited barrier was the need to obtain permission from the partner, mentioned by 8.67% of women. This obstacle may be linked to cultural and social factors, where women's health decisions are sometimes influenced by their partner's agreement. Targeted awareness campaigns emphasizing the importance of child health and the necessity for both parents to be involved in health decisions could alleviate this situation. The perceived lack of effective treatment for SCD was also a barrier for 5% of participants. Although the absence of immediate curative treatment can pose a challenge, it is crucial to acknowledge that early detection allows for disease management, reducing complications and enhancing affected children's quality of life, even without curative treatment available. Some participants expressed fear of knowing their baby's sickle cell status (3%) or apprehension that the injection might harm their child (1.67%), emphasizing the importance of reassuring families about the safety and benefits of NBS. Lastly, a few women (1.67%) deemed NBS unnecessary because of their and their spouse's HbAA genotype, highlighting the necessity to educate the population better about genetic risks and the potential for having affected children even with parents who do not appear to be carriers. Addressing these concerns in future information campaigns, emphasizing the long-term benefits of NBS, even in the absence of immediate curative treatment, would be appropriate.

In developed countries, NBS with appropriate follow-up has reduced mortality from the disease from 16% to less than 1% through early diagnosis and appropriate management [30, 31]. In developing countries, however, a significant proportion of children with SCD die without appropriate diagnosis or treatment [32]. Our results are similar to those observed in other countries with a high prevalence of SCD, where NBS programs have led to a significant improvement in the management of children, increasing their life expectancy through early medical follow-up and access to quality care. These results underline the urgent need for health authorities to implement a national NBS policy in the DRC. Despite the economic and organizational challenges, including the limited knowledge among healthcare workers and the scarcity of advanced diagnostic resources as highlighted in recent studies [33], such a program could significantly improve early detection and care for affected children. By addressing these gaps in healthcare knowledge and resources, NBS has the potential to reduce mortality associated with SCD, even in a context of limited resources.

This study has some limitations that need to be considered when interpreting its findings. Firstly, the sample only includes pregnant women from Bukavu, limiting the generalizability of the results to the entire country, especially in rural areas with different healthcare access. In addition, although the sample size is representative, it may not encompass all regional or socioeconomic variations associated with NBS acceptability. Secondly, the reliance on self-reported data could introduce reporting bias, particularly regarding participants' knowledge and perceptions of SCD. The overestimation of knowledge about SCD could have occurred if some respondents were unsure and guessed their answers, which may impact the accuracy of the results related to disease awareness. Furthermore, the study did not examine practical NBS aspects like geographic accessibility, healthcare facility availability, and costs, which are crucial for NBS adherence. While sociocultural barriers like spousal involvement in decision-making were mentioned, other contextual factors such as cultural or religious beliefs were not explored in depth.

Despite these limitations, the results of this study provide valuable information on the factors associated with the acceptability of NBS and highlight the need for targeted education and awareness strategies. In particular, efforts should focus on increasing knowledge about SCD as a blood disorder and its potential for diagnosis at any stage of life. Given the nature of the study, further research is needed to test whether intervening on these factors would effectively increase acceptability. Future steps could include piloting specific strategies or interventions to evaluate their impact on improving the uptake of NBS in the DRC.

## 5. Conclusion

This study provided a better understanding of the acceptability of NBS among pregnant women in Bukavu, DRC. The results show strong support for NBS (80%), with factors such as knowledge of SCD, awareness of the possibility of early diagnosis, and the absence of associated costs playing a crucial role in this acceptability. These results are in line with other studies carried out in similar contexts, confirming the importance of information and education in promoting NBS. However, barriers remain, including the need for spousal consent and concerns about the lack of immediate curative treatment. It is therefore imperative to address these concerns in future awareness campaigns, emphasizing the long-term benefits of NBS, even in the absence of curative treatment.

The public health implications of this study are manifold. It highlights the need to integrate NBS into public health policies in the DRC, particularly in areas with a high prevalence of the disease. Targeted efforts should be made to enhance education about SCD, particularly regarding early diagnosis and the long-term benefits of NBS, in order to overcome social and cultural resistance. In addition, expanding this research to other regions and examining practical aspects of NBS, such as accessibility and costs, would further support the nationwide implementation of this crucial public health intervention.

## Figures and Tables

**Table 1:** Sociodemographic characteristics of respondents.

Variable	Number (*N* = 300)	Percentage
Age		
< 25 years	78	26.0
25–29 years	103	34.3
30–34 years	65	21.7
≥ 35 years	54	18.0
Mean ± standard deviation	28.7 ± 5.7	
Marital status		
Single	50	16.7
Married	250	83.3
Educational level		
Primary	31	10.3
Secondary	135	45.0
Higher/University	134	44.7
Professional occupation		
Commerce	154	51.3
Employee	59	19.7
None	87	29.0
Religion		
Protestants/Pentecostals	125	41.7
Catholics	123	41.0
Muslims	52	17.3

**Table 2:** Acceptability of newborn screening for sickle cell disease according to sociodemographic characteristics and knowledge of respondents.

Variable	Total (*N* = 300)	Newborn screening		Crude OR [95% CI]	*p* value	Adjusted OR [95% CI]	*p* value
		Accepted (*n* = 240), *n* (%)	Not accepted (*n* = 60), *n* (%)				
Sociodemographic variables							
Age					0.285^∗^		
< 25 years	78	68 (87.2)	10 (12.8)	1.0			
25–29 years	103	78 (75.7)	25 (24.3)	0.5 [0.2–1.0]	0.081		
30–34 years	65	51 (78.5)	14 (21.5)	0.5 [0.2–1.3]	0.244		
≥ 35 years	54	43 (79.6)	11 (20.4)	0.6 [0.2–1.5]	0.355		
Marital status							
Married	250	196 (78.4)	54 (21.6)	1.0		1.0	
Single	50	44 (88.0)	6 (12.0)	2.0 [0.8–5.0]	0.175	2.4 [0.9–6.2]	0.068
Level of education					0.197^∗^		
Primary	31	21 (67.7)	10 (32.3)	1.0		1.0	
Secondary	135	110 (81.5)	25 (18.5)	2.1 [0.9–5.0]	0.148	1.9 [0.7–4.7]	0.180
Higher/University	134	109 (81.3)	25 (18.7)	2.1 [0.9–4.9]	0.154	1.6 [0.6–4.2]	0.326
Professional occupation					0.115^∗^		
Commerce	154	116 (75.3)	38 (24.7)	1.0		1.0	
None	87	74 (85.1)	13 (14.9)	1.9 [0.9–3.7]	0.107	1.6 [0.8–3.4]	0.184
Employee	59	50 (84.8)	9 (15.2)	1.8 [0.8–4.0]	0.194	1.4 [0.6–3.3]	0.472
Religion					0.210^∗^		
Muslim	52	37 (71.2)	15 (28.8)	1.0			
Catholic	123	100 (81.3)	23 (18.7)	1.8 [0.8–3.7]	0.198		
Protestants/Pentecostals	125	103 (82.4)	22 (17.6)	1.9 [0.9–4.0]	0.141		
Items related to SCD/NBS knowledge							
Do you know your electrophoretic status?							
No	272	216 (79.4)	56 (20.6)	1.0			
Yes	28	24 (85.7)	4 (14.3)	1.6 [0.5–4.7]	0.585		
SCD is a blood disorder							
No	92	62 (67.4)	30 (32.6)	1.0		1.0	
Yes	208	178 (85.6)	30 (14.4)	2.9 [1.6–5.1]	0.0005	2.6 [1.4–4.9]	0.002
SCD can be diagnosed in the neonatal period							
No	87	63 (72.4)	24 (27.6)	1.0		1.0	
Yes	213	177 (83.1)	36 (16.9)	1.9 [1.0–3.4]	0.036	1.6 [0.8–3.0]	0.141
SCD can be diagnosed at any other time in life							
No	89	63 (70.8)	26 (29.2)	1.0		1.0	
Yes	211	177 (83.9)	34 (16.1)	2.1 [1.2–3.9]	0.009	2.0 [1.1–3.8]	0.024

*Note:* Variables with an overall *p* value of less than 0.2 in the bivariate chi-square analysis were included in the multivariable logistic regression model. Based on this criterion, age, religion, and knowledge of electrophoretic status were excluded from the multivariable logistic regression model.

Abbreviations: 95% CI, 95% confidence interval; NBS, newborn screening; OR, odds ratio; SCD, sickle cell disease.

^∗^Overall *p* value for categorical variables with more than two modalities.

## Data Availability

The datasets used and analyzed during the current study are available from the corresponding author upon reasonable request.

## Nomenclature

95% CI: 95% confidence intervals
DRC: Democratic Republic of the Congo
Hb: Hemoglobin
NBS: Newborn screening
OR: Odds ratio
SCD: Sickle cell disease
SSA: Sub-Saharan Africa

## Supporting Information

The supporting file contains the STROBE checklist, providing a detailed account of how our manuscript adheres to the Strengthening the Reporting of Observational Studies in Epidemiology (STROBE) guidelines.
